# Effects of Age on Female Reproductive Success in *Drosophila bipectinata*

**DOI:** 10.1673/031.011.13201

**Published:** 2011-10-04

**Authors:** K Somashekar, Ms Krishna, Sn Hegde, SC Jayaramu

**Affiliations:** *Drosophila* Stock Center, Department of Studies in Zoology, University of Mysore, Manasagangotri, Mysore 570006, Karnataka State, India

**Keywords:** male preference, wing length, female age

## Abstract

Female age influence on mating success, courtship activities, mating latency, copulation duration, fecundity, ovarioles number, and wing length has been studied using isofemale lines of *Drosophila bipectinata* collected at three different localities. It was observed that in all localities, middle-aged *D. bipectinata* females had significantly greater mating success, showed less rejection responses to courting male, mated faster, copulated longer, and had greater fecundity and ovariole number than young and old-aged females. Further, old-aged females had comparatively less fitness traits than young age females. This research suggests the occurrence of age specific female reproductive success as follows: middle-aged > young > old-aged.

## Introduction

Models of sexual selection predict that females are more selective because of their greater parental investment (Bonduriansky 2001). Although mate choice in males is rare, the phenomenon has been reported in 58 insect species distributed among 11 orders and 37 families (Bonduriansky 2001). Male choice has been reported in a few species of birds (Jones and Hunters 1993) and fishes (Amundsen and Forsgren 2001). The high cost of reproduction in males of many species has become increasingly apparent; influencing factors include energetically expensive courtship displays (Judge and Brooks 2001) and the production of ejaculates (Dewsbury 1982; Galvani and Johnstone 1998). Individuals selecting their mating partners are likely to lose mating opportunities and energy in search for more attractive suitors. In order for this behavior to be adaptive, individuals must be expected to benefit one way or another (Trivers 1972). Benefits may come in the form of direct enhancement of survival or fecundity; selection favors mating preferences towards mates that are more fertile, provide superior resources, offer more parental care, or otherwise help to reduce reproductive costs (Andersson 1994). On the other hand, selective mating may also be adaptive as a consequence of indirect benefits; the offspring may inherit genes that promote their survival or reproduction (Andersson 1994). Specifically, mate choice may lead to production of offspring with genotypes that increase viability, or that make them more attractive to the opposite sex.

The most compelling studies of male choice suggest that female mating success is often associated with traits that are correlated with female fecundity (Bonduriansky 2001; Byrne and Rice 2006). However, models of the evolution of male choice suggest that male choice will tend to breakdown when males target arbitrary female traits rather than those that reliably signal fecundity (Kokko and Johnstone 2002; Chenoweth et al. 2006; Servedio and Lande 2006). Therefore, male preference for female traits, such as body size or age, may be an indirect way of assessing female fecundity. Insects studies have shown influence of age on mating success, activity, and reproductive performance (Eberhard 1996; Byrne and Rice 2006). Recently in *D. ananassae,* Prathibha and Krishna (2010) have found greater mating success in middleaged females compared to young and old-aged females, suggesting that female age, rather than female body size, is correlated with female reproductive success in *Drosophila.* However, Prathibha and Krishna (2010) used only one strain for their study; more studies are necessary in *Drosophila*—and insects in general—to build understanding of age-specific reproductive performance in females.

*D. bipectinata* belongs to the *bipectinata* complex of *ananassae* subgroup of the *melanogaster* species group. *D. bipectinata* is a wild species, commonly occurring in the Indian sub-continent. This species has attracted the attention of local researchers during the past few years, resulting in extensive studies on its populations and behavioral genetics. Males of *D. bipectinata* show many similarities in behavior with other *Drosophila,* including lack of parental care, contribution of sperm and components of ejaculate to the courted female (Hegde and Krishna 1997), high reproduction costs due to factors such as energetically expensive courtship displays, production of ejaculates, and time loss during courtship displays (Krishna 1998). Therefore, our study on *D. bipectinata* aims to study whether or not males of *D. bipectinata* choose females on the basis of age, and if so, what benefits are obtained. Three different strains of *D. bipectinata* were used in this study to determine if male mate choice for female age is independent of different localities or not.

## Materials and Methods

### Establishment of experimental stock

Experimental stocks established from single isofemale lines of *D. bipectinata* were collected from Dharwad, Bellur, and Mysore in August 2007. Progenies of these stocks were cultured separately, with 40 flies (20 males, 20 females) per quarter-pint milk bottle (250 ml). Bottles contained wheat cream agar medium and were maintained at 21 ± 1° C, 70% relative humidity, and 12:12 L:D. Fourth-generation synchronized eggs (± 30 minutes) were collected separately from each of the above stock using Delcour's procedure (Delcour 1969). Eggs (100) were seeded in a vial containing wheat cream agar medium. Virgin females and males were isolated within 3 hours of eclosion and were aspirated to a new vial containing wheat cream agar medium. The sexes were kept in isolation until use for the experiments.

### Selection of female age classes

Sexually mature *Drosophila* females employ a wide range of behaviors to thwart unwanted advances of courting males, such as decamping, wing flicking, kicking, and ovipositor extrusion (Spieth 1952). Newly emerged (immature) females do not perform any of these rejection behaviors (Manning 1961). Further, immature females are characterized by un-hardened cuticles, folded wings, and slow movements..

Reproductive activities of females were studied before age classes were assigned. Observations showed that females were unreceptive on the day of eclosion, and show no courting behavior toward males. From days 2–32, females were receptive and showed rejection responses such as decamping, ignoring, wing flicking, and kicking. These female behaviors began to decline after 32 days, and mating rarely occurred whatsoever after day 35.

Our experiment considered females 2–32 days old. Because females typically take 15–16 days to lay eggs and remate, three age classes were created, each separated by 15 days: young (2–3 days); middle-aged (17–18 days); and old-aged (32–33 days). For mating purposes, females aged 32–33 days were considered old-aged, though longevity of *D. bipectinata* females is 60 ± 3 days. Flies of these three age classes were collected from same culture bottle sequentially into the three age classes and were separately maintained under uniform environmental conditions. Additionally, unmated 5–6 day-old males were maintained individually in the same laboratory conditions.

### Female age influence on mating probability

To study female age influence on mating success, two females (young + middle, young + old, middle + old) and one 5–6 day-old male were individually aspirated into a mating chamber (Elens-Wattiaux 1964). Indian ink was painted on the young female in one trial, and the middle/old-aged females in alternate trials. Each pair was observed for one hour. When mating occurred, pairs in copulation were aspirated out of the mating chamber and into a new vial containing wheat cream agar medium. A total of 50 trials were run for each combination (young + middle, young + old, middle + old). Experiments were done separately for all the three strains of *D. bipectinata* studied, and Chi-square analysis was carried out on the mating success data.

### Female age influence on female mating activities and fecundity Female age influence on mating activity.

One male (5–6 days old) and one female (young/middle-aged/old-aged) were individually aspirated into a mating chamber (Elens-Wattiaux 1964) and observations were made for one hour. Female mating latency (time between introduction and initiation of copulation) and female copulation duration (time between initiation of copulation and termination of copulation) were recorded for each pair. We also quantified courtship acts such as tapping, scissoring, vibration, licking, circling, ignoring, extruding, and decamping, following the procedure of Hegde and Krishna (1997). These courtship acts are described as follows:

**Tapping:** The male initiates courtship with a foreleg motion partially extending and simultaneously elevating one or both forelegs, followed by a downward striking motion, thus bringing the ventral surface of the tarsus in contract with the partner.

**Scissoring:** The courting male opens and closes both wings with a scissor like movement during the interval between wing vibrations.

**Vibration:** The male expands one wing laterally from the resting position, and then moves one or both wings rapidly up and down.

**Licking:** The courting male positions himself closely behind the female, extends his proboscis, and licks her genitalia.

**Circling:** After posturing at the side or rear of a non-receptive female, the male faces the female as he moves about. The male may move to face her and then retraces his path to the rear, or may at other times move completely around her in a circle.

**Ignoring:** The non-receptive female simply continues with whatever activity in which she was previously engaged, apparently ignoring actions by the male.

**Extruding:** The non-receptive female presses the vaginal plates together, contracting certain abdominal muscles and apparently relaxing others.

**Decamping:** The non-receptive female attempts to escape by running, jumping, or flying away from the courting male.

Two different observers recorded the behavior of the male and female simultaneously for one hour. The number of pairs mated was also recorded.

### Female age influence on fecundity

Soon after mating, females were transferred into fresh vials containing wheat cream agar media every 24 hr to study fecundity. This continued for 32 days. Total number of eggs laid by each female was recorded. A total of 50 successfully mated pairs studied for each of the female age classes. Experiments were done separately for each of the three localities.

Two-way ANOVA and Tukey's honest post hoc tests were used on mean data of mating latency, courtship activities, copulation duration, and fecundity using SPSS 10.1 software.

A scree plot in principle component analysis indicates descending order of magnitude of the eigenvalues (In statistics, the concept of an eigenvalue is used in factor analysis to determine how many underlying factors can be extracted from a data set) of a correlation matrix. In the context of factor analysis or principal components analysis, a scree plot helps the analyst visualize the relative mimportance of the factors, a sharp drop in the plot signals that subsequent factors are ignorable.

### Female age influence on ovariole number and female wing length

In another experiment, virgin young/middleaged/old-aged females were killed to count number of ovarioles and measure female wing length following the procedure of Krishna and Hegde (1997). To count ovariole number, each female was dissected in a drop of physiological saline using a binocular stereomicroscope. Ovarioles were separated from each other from the left ovary with the help of fine needles. Number of Ovarioles in each female was counted. A total of fifty flies were used separately for each of the three female age classes. Experiments were made separately for all the three localities studied.

Two-way ANOVA and Tukey's Honest post hoc was used on wing length and ovariole number.

## Results

### Female age influence on mating probability Effects of paint on mating probability.

Before studying female age influence on mating success, the effects of paint on mating success had to be identified. Before commencing the male mate choice experiment the thorax of one of the two competing young/middle-aged/old-aged females was painted with India ink. Females were then allowed to mate, and results showed no significant difference in mating success, suggesting that paint had no influence on the performance of the flies (Table 1).

Table 2a revealed that males generally chose to mate with middle-aged females more frequently than young or old-aged females. Mating success of middle-aged females (N = 50) was 82% in Mysore, 80% in Dharwad, and 84% in Bellur in crosses involving young and middle-aged females. Similarly, mating success of middle-aged females (N = 50) was 80% in Mysore, 74% in Dharwad, and 76% in Bellur in crosses involving old and middleaged females. In crosses involving young and old-aged females (N = 50) mating success of young females was 60% in Mysore, 52% in Dharwad, and 56% in Bellur.

Logistic regression was also applied on female mating success data, which showed significant differences between female age classes (Table 2b). Males chose middle-aged females more than either young or old-aged females.

### Age influence of female mating activities and fecundity

**Figure 1.**
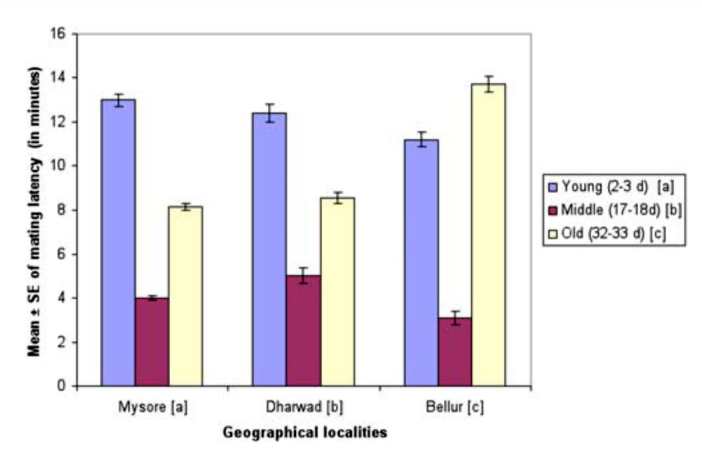
Female age influence on mating latency in different geographical localities of *Drosophila bipectinata.* Different alphabet letters in parentheses indicate significant variation by Tukey's post hoc test. 50 trials were made separately for each of the young, middle, and old-aged females. Experiments were conducted separately for all the three geographical localities. High quality figures are available online.

Mean values of time taken for mating of young, middle-aged, and old-aged females is provided in Figure 1. Lowest mean mating latency was found in Bellur, while flies in Mysore took the longest time for mating. Among female age classes, middle-aged females took the least time for initiation of copulation, while old-aged females' mating latency was longest. Two-way ANOVA and Tukey's post honest post hoc test (Tukey's test) used in analysis of mating latency data (Table 3) showed significant variation between female age classes among localities, and also showed an interaction between locality and female age classes. Tukey's test showed that middle-aged females took significantly less time for mating when compared to young or old-aged females.

**Figure 2.**
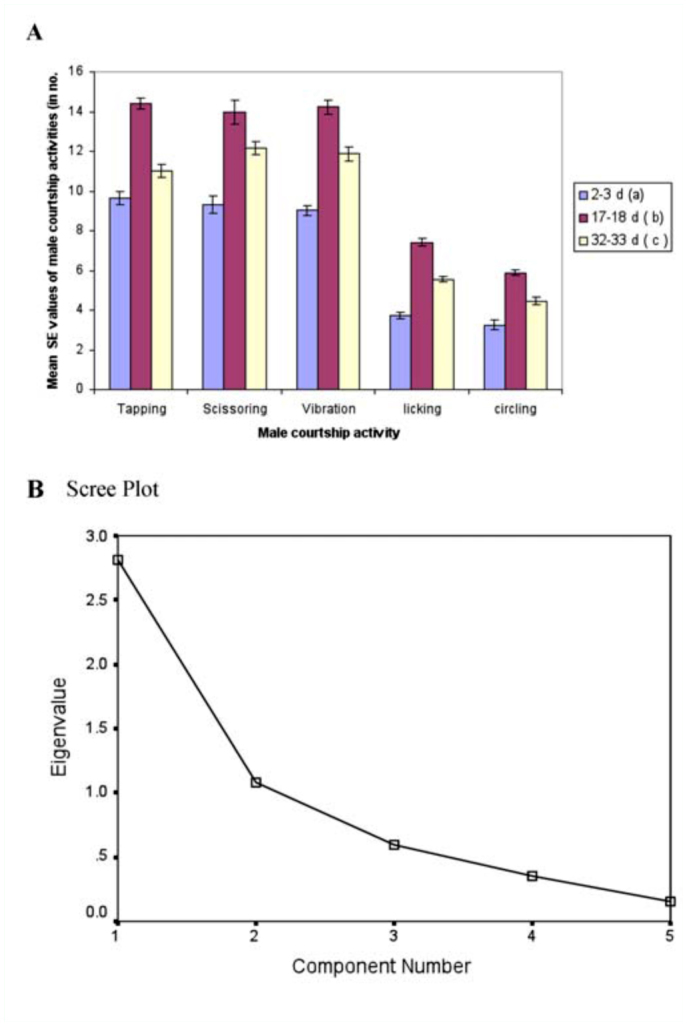
A). Female age influence on male courtship activities (on pooled data) in *Drosophila bipectinata.* Different alphabet letters in parentheses indicate significant variation by Tukey's post hoc test. 150 trials (from three localities) were made separately for young, middle, and old-aged females. B). Scree plot of principle component analysis for male courtship activities in *D. bipectinata.* High quality figures are available online.

Mean male courtship acts directed toward young, middle-aged, and old-aged females are provided in Figure 2a. It was observed that males' courtship acts (i.e., tapping, scissoring, vibration, licking, circling) were directed most frequently toward middle-aged females, while far less courting was directed to young females. One-way ANOVA carried out on pool data (Table 4) of all localities showed significant variation in male courtship acts between different female age classes. Tukey's post hoc test also showed male courtship acts toward middle-aged females was significantly greater when compared to male courtship acts toward old-aged or young females. Principle component analysis applied on male courtship acts toward females of different age classes showed that among the courtship acts, tapping and scissoring had the greatest influence on female mating success compared to vibration, licking, and circling (Table 5, Figure 2b). This was also evident in a scree plot of male courtship acts toward young, middle-aged, and old-aged females.

**Figure 3.**
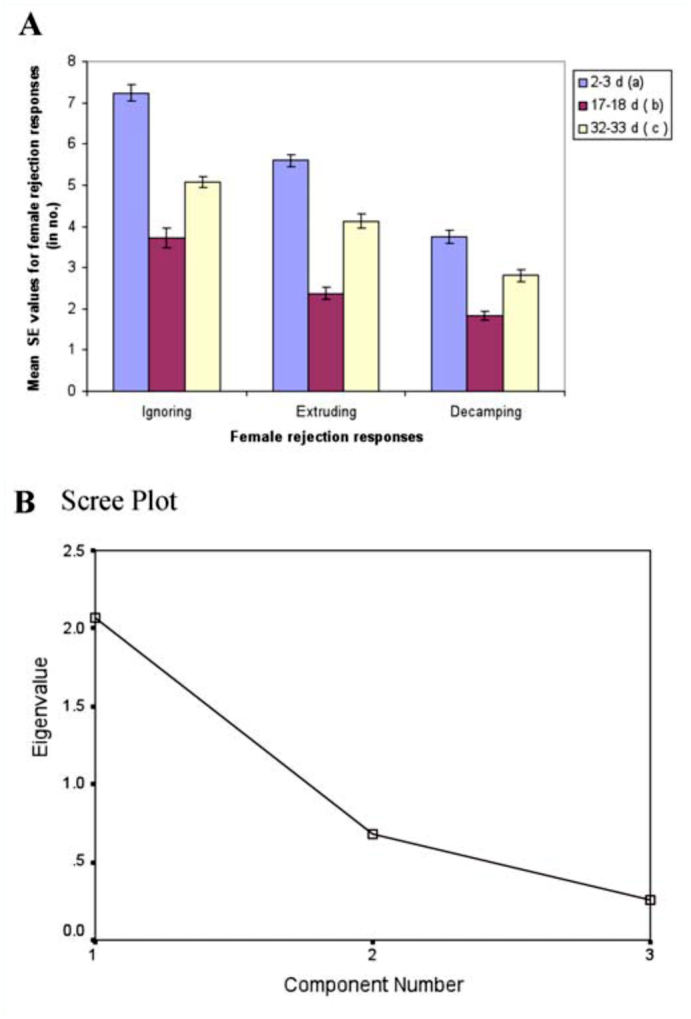
A). Female age influence on female rejection responses (on pooled data) in *Drosophila bipectinata.* Different alphabet letters in parentheses indicate significant variation by Tukey's post hoc test. 150 trials (from three localities) were made separately for young, middle, and old-aged females. B). Scree plot of principle component analysis for female rejection responses in *D. bipectinata.* High quality figures are available online.

**Figure 4.**
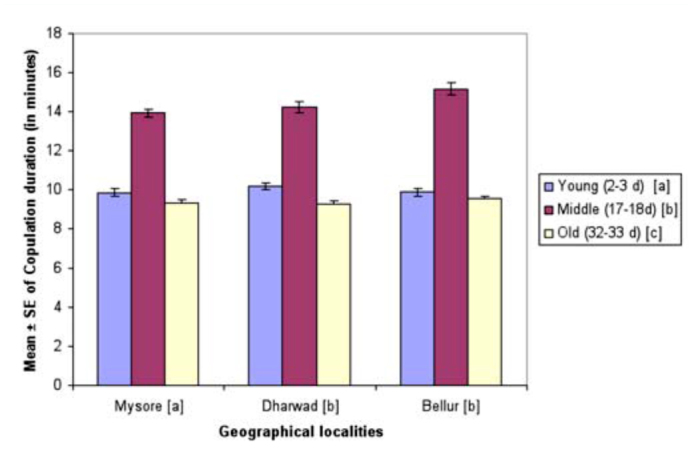
Female age influence on copulation duration in different geographical localities of *Drosphila bipectinata.* Different alphabet letters in parentheses indicates significant variation by Tukey's post hoc test. High quality figures are available online.

Figure 3a shows mean female rejection responses of young, middle-aged, and old-aged females to courting males. Middle-aged females showed the lowest rejection response (i.e., ignorance, extruding, decamping) to courting males, while young females showed least rejection responses to courting male. One-way ANOVA and Tukey's post hoc test showed significant variation in female rejection responses between female age classes (Table 6). Tukey's test also showed middle-aged females showed significantly less rejection response compared to old-aged or young females. The principle component analysis carried on female rejection responses to courting males showed that females' ignoring and extruding acts toward courting males was found to be a greater influence on female mating success than female decamping acts (Table 7, Figure 3b).

**Figure 5.**
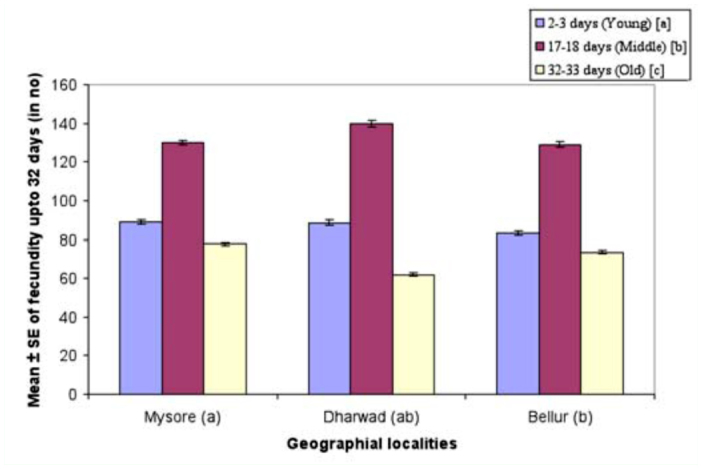
Female age influence on life time fecundity (32 days) in different geographical localities of *Drosophila bipectinata.* Different alphabet letters in parentheses indicate significant variation by Tukey's post hoc test. 50 trials were made separately for each of the young, middle, and old-aged females. Experiments were conducted separately for all the three geographical localities. High quality figures are available online.

Female age influence on copulation duration is shown in Figure 4. Flies in Bellur copulated the longest while flies in Mysore copulated for the shortest duration of time. In female age classes, middle-aged females copulated longest whereas young female flies copulated for the shortest duration of time. Copulation duration data of young, middle-aged, and old-aged females was subjected to two-way ANOVA followed by Tukey's post hoc test (Table 8). This analysis showed significant differences between female age classes across localities, and also showed an interaction between localities and female age. Middle-aged females copulated for significantly longer durations of time compared to young or old-aged females. Of all localities, flies in Mysore showed the shortest copulation time.

Figure 5 shows female age influence on fecundity in *D. bipectinata.* Fecundity was highest in Bellur and lowest in Mysore. Fecundity was found to be highest in middle-aged females and lowest in old-aged females. Two-way ANOVA and Tukey's test (Table 9) were used to analyze fecundity data of young, middle-aged, and old-aged females. Results showed significant differences in fecundity between female age classes, between strain of *D. bipectinana,* and also showed an interaction between strains and female age.

**Figure 6.**
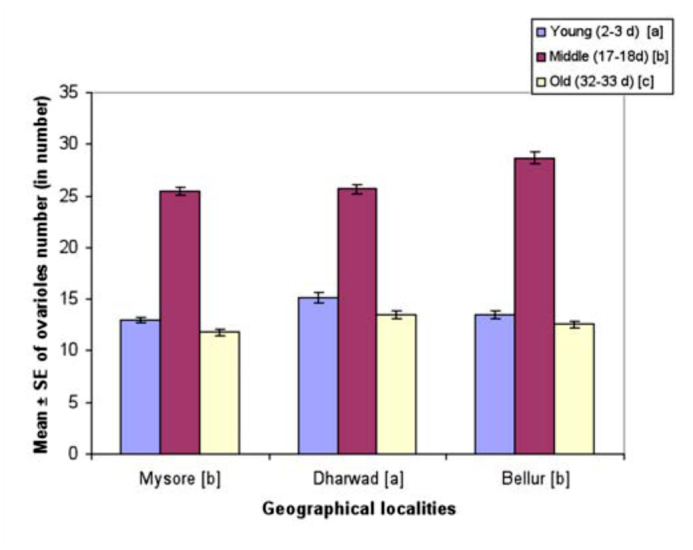
Female age influence on ovarioles number in different geographical localities of *Drosophila bipectinata.* Different alphabet letters in parentheses indicate significant variation by Tukey's post hoc test. 50 trials were made separately for each of the young, middle, and old-aged females. Experiments were conducted separately for all the three geographical localities. High quality figures are available online.

### Female age influence on ovariole number and female wing length

Figure 6 shows mean ovariole number of young and old-aged females in *D. bipectinata.* Ovariole number was highest in Bellur and least in Mysore. Middle-aged females had the highest ovariole number while old-aged females had the lowest. Ovariole data was subjected to two-way ANOVA followed by Tukey's post hoc test (Table 10). Results showed significant variation in ovariole number between different female age classes, between localities, and also revealed an interaction between female age classes and localities.

**Figure 7.**
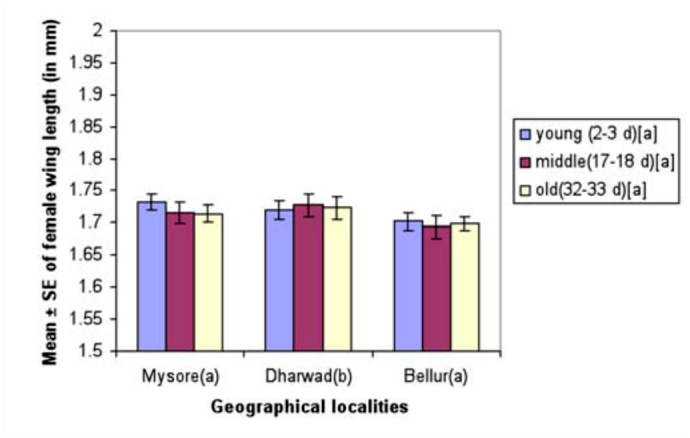
Female age influence on wing length (mm) in different geographical localities of *Drosophila bipectinata.* Different alphabet letters in parentheses indicate significant variation by Tukey's post hoc test. High quality figures are available online.

Mean wing length of young, middle-aged, and old-aged females is provided in Figure 7. Results show that mean wing length did not vary significantly by two-way ANOVA and Tukey's test (Table 11). Mean wing length of young, middle-aged, and old-aged females from all localities are provided in Figure 7. Mean female wing length varied significantly between localities but showed no significant difference between female age classes. Additionally, analysis showed an interaction between localities and female age classes.

## Discussion

Table 2a and 2b show that males of *D. bipectinata* preferred to mate with middle-aged females more frequently than young or old-aged females. This suggests that males of *D. bipectinata* show preference to in selection of females based on age, supporting earlier studies of male mate choice of female characteristics in other insects (Dewsbury 1982; Kokko and Johnstone 2002; Gowaty et al. 2003). Like the female mate choice experiment, the male mate choice experiment not only accounted for female preference but also involved female-female competition. Therefore in the choice experiment it was difficult to separate between choice and intermate competition. It seems that in *D. bipectinata* middle-aged females were more eager to mate than young or old-aged females (Table 2a, 2b). This supports the existence of sexual selection in *Drosophila* (Speith 1952; Manning 1961; Hegde and Krishna 1997; Noor 1997). These studies also suggested that successful mating in *Drosophila* might also depend on female and male courtship activities. In our study, females of different age classes were virgins, cultured and maintained in identical conditions. Therefore, the observed male preference for middle-aged females could not account for differences in female mating history.

The male mate choice experiment was not simply a measure of male preference; successful copulation also requires the target female be receptive. Past studies of *Drosophila* have suggested that male activity and female receptivity are important for successful mating (Manning 1961). Mating latency (time from courtship to copulation) is a good estimate of sexual receptivity of females and sexual activity of males (Spieth and Ringo 1983). During this period, males perform various courtship acts such as tapping, scissoring, vibration, and circling to increase the receptivity of females (Manning 1961; Spieth 1968). Figure 1 and Table 3 reveal that middle-aged female mating latency was much shorter than young or old-aged females, suggesting female age as an influence on this mating factor. Flies that took more time for mating were slow maters, while those those that took less time for mating were fast maters. Therefore, our study shows middle-aged females were faster maters compared to young or old-aged females.

We also quantified the male and female courtship activities such as tapping, scissoring, vibration, circling, licking, ignoring, extruding, and decamping in single female trials (no choice method). It was observed that males showed more courting activity toward middle-aged females compared to young or old-aged females, suggesting influence of female age on male courtship activities (Figure 2a, Table 4). In turn, middle-aged females showed less rejection responses (ignoring, extruding, decamping) to courting males than young or old-aged females, suggesting greater acceptance of males by middle-aged females (Figure 3a, Table 6). This supports earlier studies of age influence on courtship activities in different species of *Drosophila* (Speith 1952, 1968; Kokko 1997; Hegde 1979; Hegde and Krishna 1997). Through courtship activities, males convey chemical, auditory, and visual signals to middle-aged females in an attempt to convince the middle-aged female to mate. This supports earlier studies showing that males who displayed more courtship activity were better mates and obtained greater mating success than males who did not show the same high level of courtship activity (Hegde and Krishna 1997), and the importance of mating age on mating activities (Eberhard 1996, Hegde and Krishna 1997). In our study, it seems middle-aged females are better mates than young or old-aged females.

Principle component analysis showed that among male courtship acts, tapping and scissoring greater influence on female mating success compared to circling, licking, and vibration (Table 5, Figure 2b). Similarly, the female courtship act-namely ignoring and extruding—had greater influence on female mating success than decamping (Table 7, Figure 3b).

Courtship activities in *Drosophila* culminate in copulation (Spiess 1970). These activities are known to be influenced by genotype, environmental factors, male size, female size, male and female age, and strain (Guru Prasad et al. 2008). Figure 4 and Table 8 show males that mated with a middle-aged female copulated longer compared to a male that mated with young or old-aged females. Further, middle-aged females had significantly greater ovariole number and fecundity (Figure 5, 6; Table 9, 10) compared to young or old-aged females. No significant variation in female wing length was observed between females of different age classes (Figure 7, Table 11). Similar results were found in all three localities studied. This suggest that middle-aged *D. bipectinata* females have greater mating success compared to young and old-aged females. In *Drosophila* it was noticed that female reproductive physiology (i.e., egg laying) changes as females age. Egg laying was higher in middle-aged females compared to young and old-aged females (Rogina et al 2007). Additionally, age specific expression of genes and secretion of sex pheromones may also influence the mating success of middle-aged females in *Drosophila.* Therefore, the elevated mating probability in middle-aged *D. bipectinata* females could be attributed to physiological changes associated with aging.

Mating latency, copulation duration, female wing length, fecundity, and ovariole number varied significantly between different localities of *D. bipectinata* (Figure 1, 4–7; Table 3, 8–11). This supports earlier studies of intrapopulation variation of these traits in different species of *Drosophila* (Krebs 1993; Guru Prasad et al. 2008). Thus, these studies suggest age-specific female reproductive success occurs in the order as follows: middle-aged > young > old-aged.

**Table 1.**
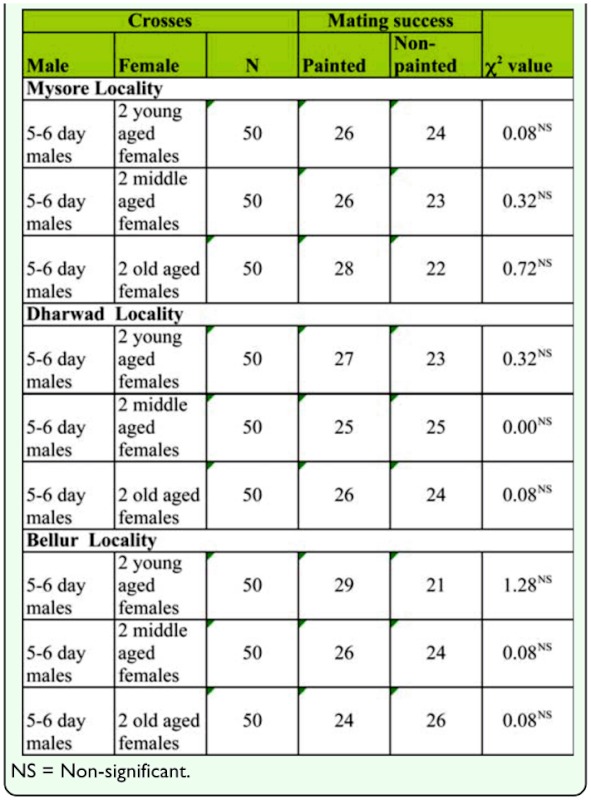
Effect of paint on mating success in three different localities of *D. bipecti.*

**Table 2a.**
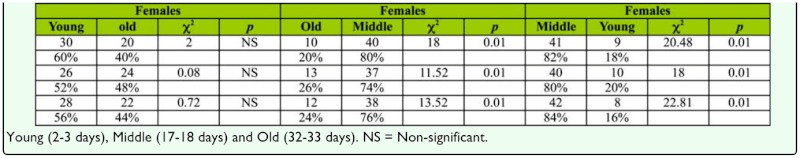
Mating success of *D. bipectinata* females from three age classes from three localities.

**Table 2b.**
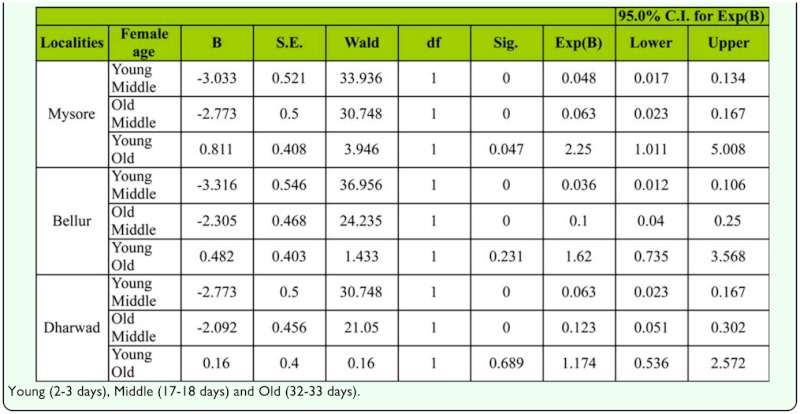
Mating success of *D. bipectinata* females from three age classes from three localities using Logistic regression.

**Table 3.**
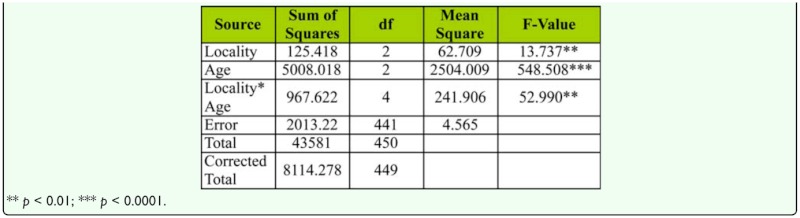
Two-way ANOVA of female age influence on mating latency in different geographical localities of *D. bipectinata.*

**Table 4.**
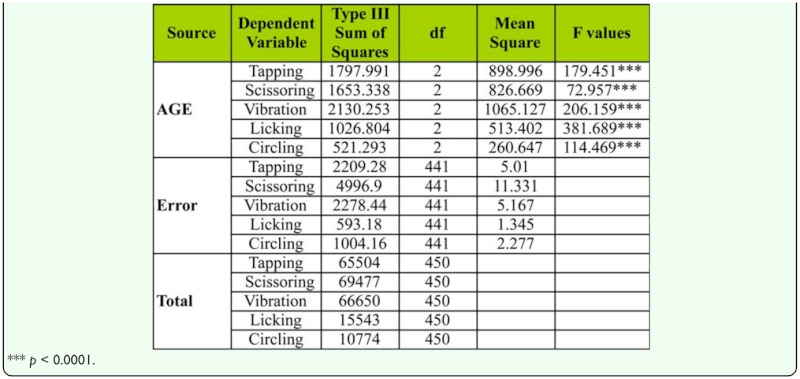
One-way ANOVA of female age influence on male courtship activities (on pool data) in *D. bipectinata.*

**Table 5.**
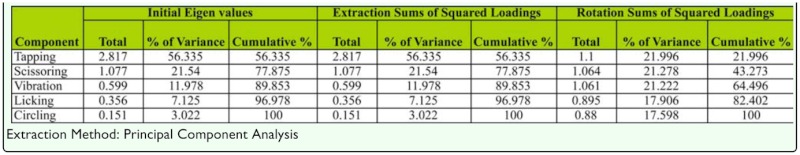
Principle component analysis for male courtship activities in *D. bipectinata.*

**Table 6.**
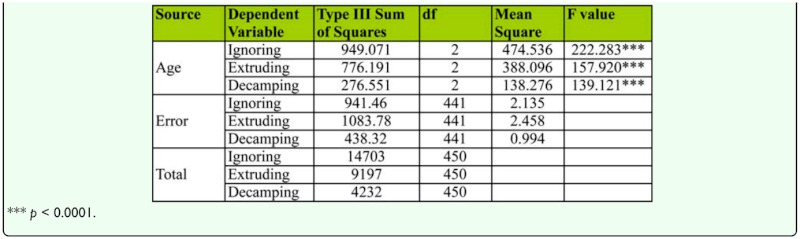
One-way ANOVA of female age influence on female rejection responses (on pool data) in *D. bipectinata.*

**Table 7.**
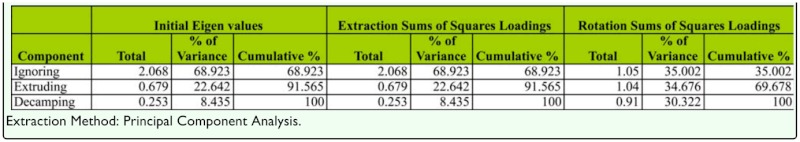
Principle component analysis for female rejection responses in *D. bipectinata.*

**Table 8.**
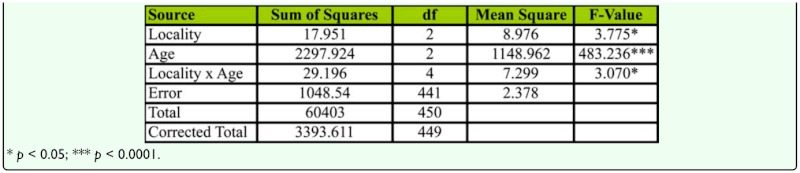
Two-way ANOVA of female age influence on copulation duration in different localities of *D. bipectinata.*

**Table 9.**
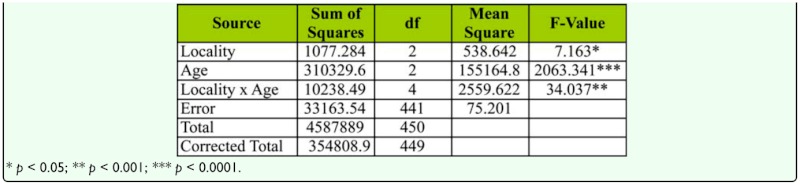
Two-way ANOVA of female age influence on life time fecundity (32 days) in different localities of *D. bipectinata.*

**Table 10.**
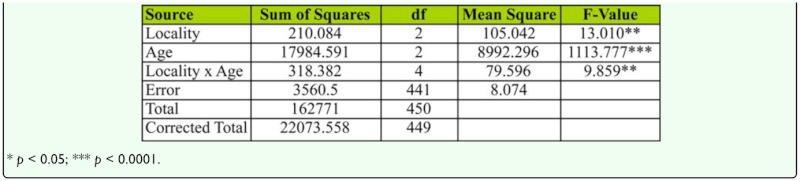
Two-way ANOVA of female age influence on ovarioles number in different localities of *D. bipectinata.*

**Table 11.**
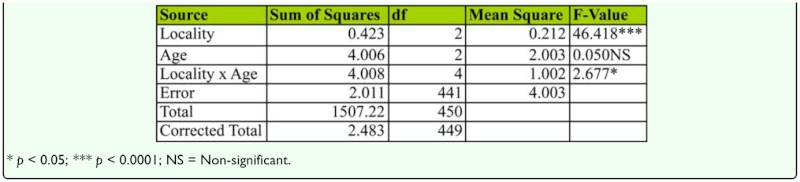
Two-way ANOVA of female age influence on wing length in different localities of *D. bipectinata*.
